# Patent Foramen Ovale Treatment Strategies Correspond to an Index Predicting Pathogenicity

**DOI:** 10.7759/cureus.4778

**Published:** 2019-05-30

**Authors:** Christopher Parr, Shuangbo Liu, Brittany Perija, Nasir Shaikh, Malek Kass

**Affiliations:** 1 Cardiology, University of Manitoba, Winnipeg, CAN; 2 Cardiology, University of Toronto, Toronto, CAN; 3 Internal Medicine, University of Manitoba, Winnipeg, CAN

**Keywords:** patent foramen ovale, stroke, cryptogenic stroke, interventional cardiology

## Abstract

Background: Percutaneous closure of patent foramen ovale (PFO) in patients with cryptogenic stroke (CS) may reduce the risk of recurrent stroke. By performing closure only in those with high risk of recurrent PFO related strokes, patient selection may be improved. The Risk of Paradoxical Embolism (RoPE) score is a point-based index developed to estimate the probability that the index CS was attributable to patent foramen ovale. We aimed to evaluate whether management strategies using conventional clinical judgement for patients with CS and PFO corresponded with RoPE scores.

Methods: We performed a single-centre retrospective chart review of adult patients with CS or transient ischemic attack who were evaluated for PFO closure from January 1, 2011 to December 31, 2017. Patients were categorized based on the treatment strategy of percutaneous closure or medical management. RoPE scores were computed and clinical outcomes evaluated.

Results: A total of 154 patients were included: 63 patients underwent percutaneous closure and 91 patients were treated medically. Mean RoPE scores for closure and medical groups were 6.9±1.5 and 4.7±1.9, respectively (p<0.001). For patients who underwent percutaneous closure, successful device delivery was achieved in all patients and there were no immediate complications.

Conclusion: In this single-centre study, patients selected for percutaneous PFO closure based on conventional clinical judgement were more likely to have elevated PFO attributable risk, based on the RoPE score.

## Introduction

Patent foramen ovale (PFO) is more prevalent in patients with cryptogenic stroke (CS) than in the general population [1]. Studies have identified a variety of potential mechanisms by which PFO may lead to ischemic stroke [2-3], the most prominent being paradoxical emboli. Given this theoretical causality, it has been proposed that PFO closure may lead to a reduction in morbidity and mortality. Recent trials including CLOSE [4], REDUCE [5], and the long-term outcome data from RESPECT [6] suggest lower risks of recurrent ischemic stroke for patients treated with PFO closure, yet patient selection differed between these three major studies. The prevalence of PFO in otherwise structurally normal hearts is 27.3% [7], but not all PFOs inevitably result in a stroke. With such a high general prevalence of PFO, identifying which PFO are incidental versus potentially pathogenic may help identify patients who may benefit from percutaneous closure [8].

The Risk of Paradoxical Embolism (RoPE) Score is a point-based index developed to estimate the likelihood that a PFO was related to the index CS [9]. This attributable fraction depends upon several variables that comprise age, hypertension, diabetes mellitus, current smoking, history of previous stroke or transient ischemic attack (TIA), and location of the stroke on imaging. Ranging from 0 to 10, a higher RoPE score identifies those with a higher chance that a patient’s CS is related to PFO, while simultaneously predicting lower rates of two-year stroke or TIA recurrence [9]. Those with a RoPE score greater than 6 are considered high risk with a PFO attributable fraction between 77%-83% and those with a score 6 or less are considered low risk with a PFO attributable fraction between 36%-43% [10].

Clinicians are likely to select patients for PFO closure based on their assessment of whether a patient’s CS is related to a PFO and whether the benefits of closure outweigh the procedural risks. The variables identified in the RoPE score are in keeping with clinically-intuitive markers of vascular risk [9,11-12]. Given the variety of proposed risk factors for CS apart from PFO, patient selection for closure may be heterogeneous between practitioners [13]. It is unclear whether decisions regarding PFO closure in contemporary practice correspond to this evidence-based tool for predicting pathogenic PFO. Our objective is to determine whether management strategies using conventional clinical judgement for patients with CS and PFO corresponded with RoPE scores.

## Materials and methods

All patients aged ≥18 years who were assessed for PFO closure at the St. Boniface Hospital Structural Clinic from January 1, 2011 to December 31, 2017 were screened for inclusion in our retrospective study. Patients with PFO present on transthoracic echocardiography (TTE) or transesophageal echocardiography (TEE) and whose reason for referral was CS or TIA were included. Definitions of index cerebrovascular event (CS or TIA), radiographic descriptors, and patient characteristics were consistent with the RoPE study [9,14-15] and TOAST (Trial of Org 10172 in Acute Stroke Treatment) classification [16]. Accordingly, index cerebrovascular events were classified as stroke if there were sudden-onset neurologic deficits due to cerebral ischemia lasting at least 24 hours or with relevant imaging, or as TIA if lasting less than 24 hours with unremarkable imaging. Patients were included in the closure group if they underwent PFO closure or the medical group if only medical treatment was pursued.

Baseline clinical variables and pre-procedure antithrombotic medication use were collected from hospital records. Imaging tests including echocardiography (TTE and TEE), computed tomography (CT), and cardiac magnetic resonance imaging (MRI) were collected from imaging databases. Data regarding left ventricular ejection fraction, visualization of PFO, atrial septal aneurysm, the direction of the shunt, shunting at rest (or with the Valsalva maneuver), multiple PFOs, and left atrial thrombus were collected. Cardiac MRI findings were reviewed for the presence of anomalous pulmonary venous return and porto-systemic shunting.

RoPE scores were computed for each patient based on the variables described previously [9]. Implanted devices include the Amplatzer PFO Occluder (St. Jude Medical Inc., Plymouth, MN, USA) and Gore Septal Occluder (GSO) (W. L. Gore & Associates Inc., Newark, DE, USA). Device delivery success was defined as correct positioning of device with post-procedure shunt grade of 0 on echocardiography. Patients subjected to closure had routine TTE follow-up at three months and clinical follow-up at six months.

The study design and protocol were approved by the University of Manitoba Bannatyne Campus Health Research Ethics Board and the St. Boniface Hospital Research Review Committee.

For descriptive statistics, continuous variables with normal distribution were expressed as mean and standard deviation, and non-normally distributed variables were presented as median and interquartile range. The Shapiro-Wilk test was used to assess for normality of distribution. For comparison of continuous variables between the two cohorts (closure and medical groups), t-test was used for normally distributed variables and Wilcoxon test was used for skewed variables. For categorical variables, Fisher’s exact or chi-square test was used. For ordinal variables, the Mann-Whitney U test was used. All tests were two-sided and a significance level of p <0.05 was considered to indicate statistical significance. Analyses were performed using SAS software, version 9.4 (SAS Institute Inc., Cary, North Carolina, USA). Missing data were excluded from analysis of the variable in question.

## Results

**Figure 1:**
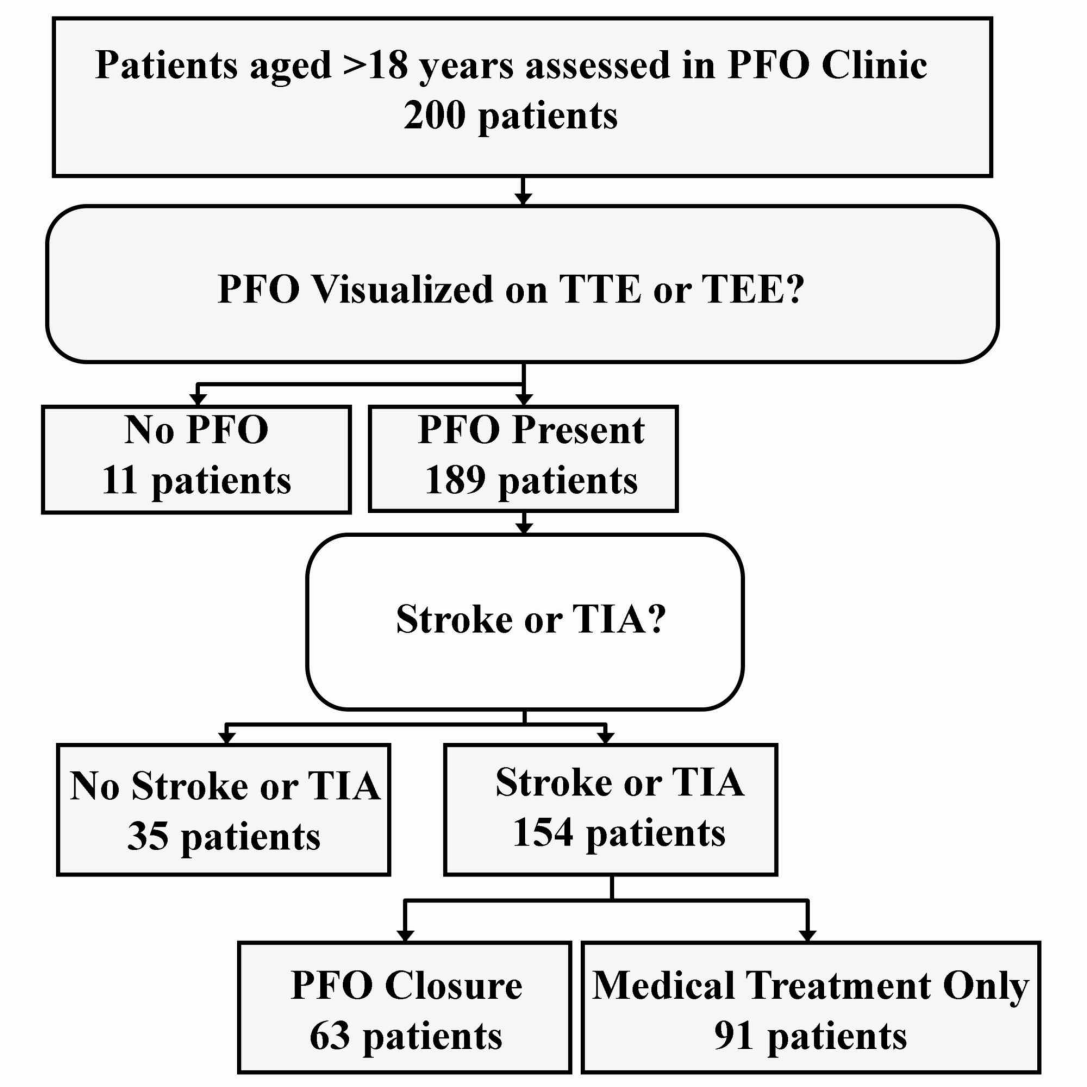
Flow-chart of Patient Inclusion and Exclusion PFO: Patent foramen ovale; TTE: Transthoracic echocardiogram; TEE: Transesophageal echocardiogram; TIA: Transient ischemic attack.

A total of 200 patients were evaluated in the structural clinic between January 1, 2011 and December 31, 2017 (Figure 1). Eleven patients were excluded because they did not have a PFO and 35 patients did not have a CS or TIA. Therefore, 154 patients were included in the analysis; 63 patients underwent percutaneous closure (closure group) and 91 patients were treated medically (medical group).

Baseline patient characteristics are shown in Table 1. Mean age was 47 ± 11 years for the closure group and 55 ± 13 years for the medical group (p <0.001). Notably, rates of hypertension, diabetes mellitus, dyslipidemia, dyspnea, history of stroke, and current smoking were lower in the closure group. Patients in the closure group were more likely to present with cortical stroke (p <0.001).

**Table 1:** Patient Characteristics

Table 1. Patient Characteristics			
Characteristic	Closure Group (n=63)	Medical Group (n=91)	p value
Age - year			
At assessment	47 ± 11	55 ± 13	<0.001
At stroke	45 ± 11	54 ± 13	<0.001
Male sex – no. (%)	36 (57%)	43 (47%)	0.227
Medical history – no. (%)			
Diabetes	3 (5%)	24 (26%)	0.001
Hypertension	17 (27%)	52 (57%)	<0.001
Current smoker	22 (35%)	53 (58%)	0.004
Prior stroke or transient ischemic attack	9 (14%)	44 (48%)	<0.001
Coronary artery disease	4 (6%)	7 (8%)	0.223
Dyslipidemia	18 (29%)	53 (58%)	<0.001
History of dyspnea	7 (11%)	22 (24%)	0.041
Antithrombotic medications – no. (%)			
ASA	36 (57%)	65 (71%)	0.067
Clopidogrel	20 (32%)	25 (27%)	0.567
Warfarin	14 (22%)	14 (15%)	0.272
Direct oral anticoagulant	4 (6%)	7 (8%)	0.750
Dalteparin	2 (3%)	1 (1%)	0.359
No antithrombotic	2 (3%)	2 (2%)	0.708
Index cerebrovascular event – no. (%)			
Cortical stroke	46 (73%)	33 (36%)	<0.001
Lacunar stroke	7 (11%)	26 (29%)	
Transient ischemic attack	10 (16%)	32 (35%)	

Left ventricular ejection fraction was preserved (>60%) in most patients for both the closure and medical groups (98% vs. 93%; p = 0.14). Those selected for closure were more likely to have evidence of atrial septal aneurysm (57% vs. 22%; p <0.001). There were no significant differences in PFO visualization on TTE (92% vs. 93%; p = 0.750), or shunt direction (p = 0.289) between the two groups. Left atrial thrombus was visualized by TEE post-referral in two patients in the medical group. Cardiac MRI was performed in 12% of patients. One patient (0.6%) had pulmonary-to-systemic flow demonstrated on MRI. There was no evidence of anomalous pulmonary venous return in any patient assessed with MRI.

**Figure 2:**
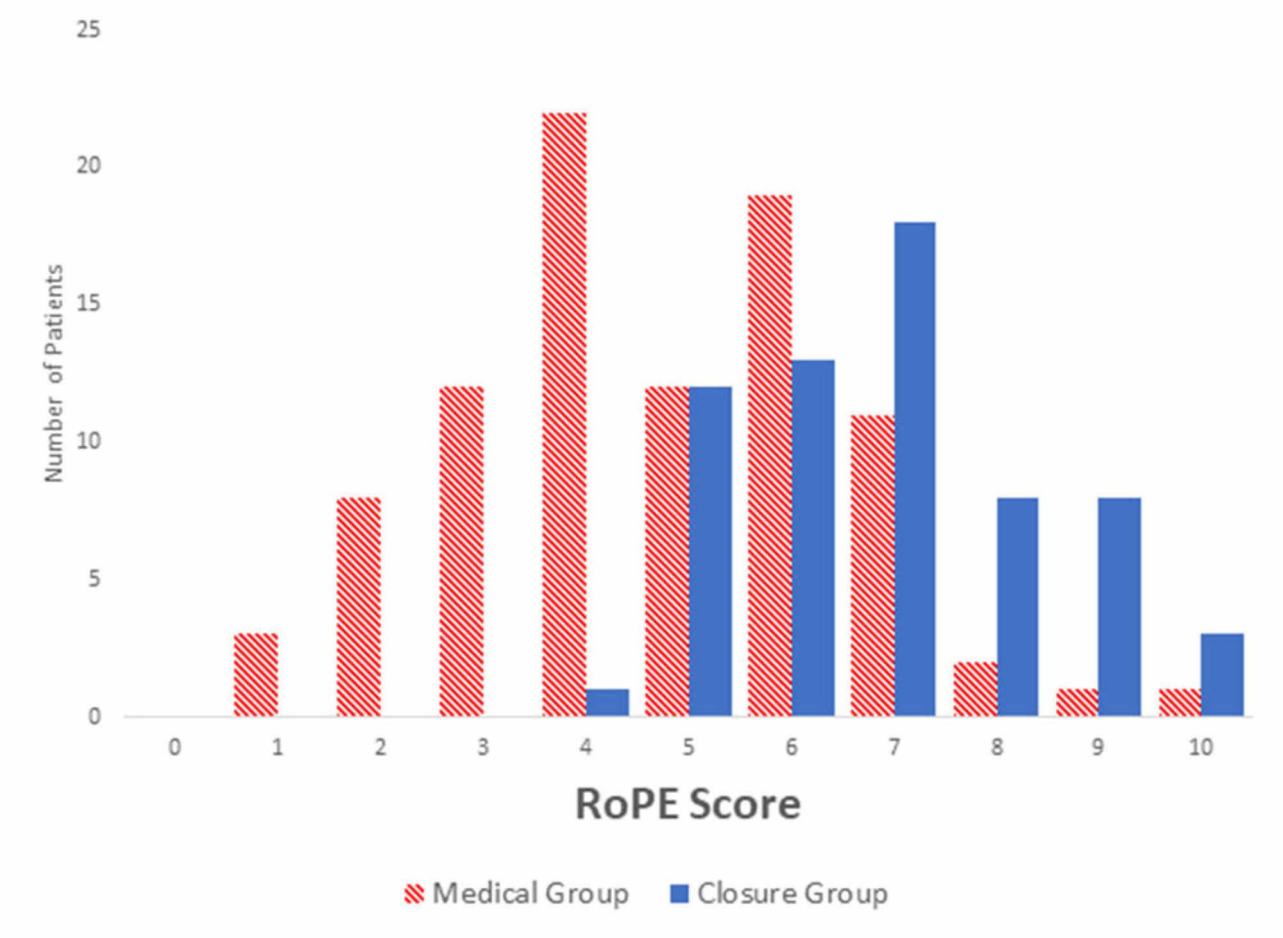
Risk of Paradoxical Embolism (RoPE) Score by Treatment Group RoPE scores were significantly different between treatment groups (p <0.001).

Mean RoPE scores for closure and medical groups were 6.9±1.5 and 4.7±1.9, respectively (Figure 2). RoPE scores were significantly different between treatment groups (p <0.001). Of those selected for closure, 59% had a RoPE score >6 compared to 16% of medically treated patients (p <0.001). There were no statistically significant differences in the percentage of assessed patients selected for closure between the periods before and after the publication of the original RoPE study (August 31, 2013). Mean RoPE scores for closure and medical management groups were similar in patients evaluated both before (closure 6.3±1.3; medical 4.6±1.6) and after (closure 7.2±1.5; medical 4.7±1.9) the date of the publication of the original RoPE study.

The Amplatzer PFO Occluder was used in two patients (3%) and the GSO device was used in the remaining 61 patients (97%). Mean device size was 24 ± 2.6 mm. Device delivery success was 100%, and no immediate complications were noted. Among patients selected for PFO closure, 46 patients (73%) were evaluated in the clinic at a mean of 6.3 months (17 patients lost to follow-up), and none had clinical or radiographic evidence of recurrent stroke or TIA. Repeat TTE was available in 62 patients (98%) at a mean of 3.7 months post-operatively: three patients (4.8%) had residual right-to-left shunt with Valsalva and there were no significant complications noted.

## Discussion

For those evaluating patients for PFO closure after CS, the RoPE study disaggregates risk into two components. The first is whether the CS is related to PFO, as estimated by the RoPE score. The second is whether a patient with pathogenic CS is at risk for further stroke or TIA. Presenting with index TIA, septal hypermobility, small shunt, and history of stroke or TIA are risk factors for repeat events [10]. Paradoxically, patients with a high likelihood of pathogenic PFO were less likely to have recurrent cerebrovascular events.

Our findings demonstrate that the selection of patients for PFO closure based on clinician discretion in our centre is in agreement with high RoPE scores. We also noted no significant differences in patient selection both before and after the publication of the RoPE study, suggesting that clinical judgement was not influenced by the publication of the RoPE score, which had not yet been externally validated for clinical use. Older patients with a higher prevalence of conventional vascular risk factors were less likely to be chosen for closure. This is consistent with supplemental data from the RoPE database [10] showing that patients were more likely to be treated with closure if they were younger or less likely to smoke. Patients selected for closure in the original RoPE database were statistically more likely to have shunt at rest (88.8% vs. 67.2%; p <0.0001) and less likely to have coronary artery disease (2.1% vs. 7.9%; p = 0.0127), suggesting these factors guided treatment decisions in the component studies. Furthermore, they were equally likely to have dyslipidemia and prior stroke or TIA. In contrast, the patient selected for closure in our study were less likely to have dyslipidemia (29% vs. 58%; p <0.001) and prior stroke or TIA (14% vs. 48%; p <0.001). This implies that clinicians at our centre considered a history of stroke and dyslipidemia as conventional vascular risk factors that would predispose to a non-PFO-related cause of stroke.

At least one major randomized trial showing benefit for PFO closure included only patients with atrial septal aneurysm or large shunt [4]. While only roughly half of patients in our study selected for closure had atrial septal aneurysms, those with atrial septal aneurysms were more likely to be selected for closure.

Studies using the RoPE score to evaluate risk factors for recurrent strokes in patients with elevated scores [10] and transesophageal markers [17] have been performed using the RoPE study database. Our report is the first published manuscript to study an external population. Baseline characteristics in our study were similar to the population of the RoPE study database. However, rates of incident TIA were higher in our study than the RoPE study. This difference may be due to differences in general patient populations, referral practices, or diagnosis. Initial presentation with a TIA was identified as a risk factor for stroke recurrence, independent of RoPE score [10]. The diagnosis of TIA may be challenging, with significant heterogeneity in diagnosis between physicians and centres [18-19]. Notably, two major studies demonstrating benefit for closure included only patients presenting with CS and not TIA [4-5].

Device delivery success rate was high, with no immediate complications associated with device implantation. Most patients selected for percutaneous closure received the GSO device. Our high device success rates are in keeping with the data reported in the literature [20-22], suggesting similar technique and operator expertise. Current guidelines regarding PFO closure in CS are largely based on studies using other devices [23], although the REDUCE study [5] has shown benefit with the GSO device. Therefore, with both improvement in patient selection afforded by the RoPE score (and ultimately a single score identifying patients at high “PFO-attributable recurrence risk” [10]) and device safety, new data may arise showing benefit in selected patients.

There are several limitations with this study. It was not powered for assessment of differences in long-term outcomes between groups. A substantial proportion of those scheduled for routine post-operative clinic evaluation after closure were lost to follow-up. This was compounded by the low rates of stroke recurrence in this population. Therefore, we were unable to determine the risk factors for recurrent stroke in our patient population and compare it to the RoPE study [10]. The prevalence of patients with cryptogenic stroke not referred to our structural clinic is unknown. Referral patterns from community stroke neurologists may be variable and exclusion of these patients implies a source of referral bias that cannot be quantified. Recognizing this, we focused specifically for evaluation of selection for closure in patients who were assessed in our clinic. Another limitation is the inconsistency of data regarding echocardiographic variables proposed to be associated with CS. Since only 75/154 (48%) patients underwent TEE, almost exclusively in the closure group, we would not have been able to compare between closure and medical groups. Yet echocardiographic data relating to PFOs are noted to be heterogeneously reported without standardized criteria on reporting PFO anatomy [10].

## Conclusions

In conclusion, patients with CS and PFO who are selected for percutaneous closure at our centre have higher PFO-attributable risk according to the RoPE score. Patients selected for closure are younger and less likely to have conventional vascular risk factors, and hence are more likely to have a pathogenic PFO.

## References

[1] Wu LA, Malouf JF, Dearani JA (2004). Patent foramen ovale in cryptogenic stroke: current understanding and management options. Arch Intern Med.

[2] Messe SR, Silverman IE, Kizer JR, Homma S, Zahn C, Gronseth G, Kasner SE (2004). Practice parameter: recurrent stroke with patent foramen ovale and atrial septal aneurysm. Neurology.

[3] Mas JL, Arquizan C, Lamy C (2001). Recurrent cerebrovascular events associated with patent foramen ovale, atrial septal aneurysm, or both. N Engl J Med.

[4] Mas JL, Derumeaux G, Guillon B (2017). Patent foramen ovale closure or anticoagulation vs. antiplatelets after stroke. N Engl J Med.

[5] Sondergaard L, Kasner SE, Rhodes JF (2017). Patent foramen ovale closure or antiplatelet therapy for cryptogenic stroke. N Engl J Med.

[6] Saver JL, Carroll JD, Thaler DE (2017). Long-term outcomes of patent foramen ovale closure or medical therapy after stroke. N Engl J Med.

[7] Hagen PT, Scholz DG, Edwards WD (1984). Incidence and size of patent foramen ovale during the first 10 decades of life: an autopsy study of 965 normal hearts. Mayo Clin Proc.

[8] Thaler DE (2014). Patient selection for PFO closure based on the RoPE study. Cardiovasc Interv.

[9] Kent DM, Ruthazer R, Weimar C (2013). An index to identify stroke-related vs incidental patent foramen ovale in cryptogenic stroke. Neurology.

[10] Thaler DE, Ruthazer R, Weimar C (2014). Recurrent stroke predictors differ in medically treated patients with pathogenic vs. other PFOs. Neurology.

[11] Alsheikh-Ali AA, Thaler DE, Kent DM (2009). Patent foramen ovale in cryptogenic stroke: incidental or pathogenic?. Stroke.

[12] Lechat P, Mas JL, Lascault G (1988). Prevalence of patent foramen ovale in patients with stroke. N Engl J Med.

[13] Messe SR, Kent DM (2013). Still no closure on the question of PFO closure. N Engl J Med.

[14] Thaler DE, Di Angelantonio E, Di Tullio MR (2013). The risk of paradoxical embolism (RoPE) study: initial description of the completed database. Int J Stroke.

[15] Thaler DE, Ruthazer R, Di Angelantonio E (2013). Neuroimaging findings in cryptogenic stroke patients with and without patent foramen ovale. Stroke.

[16] Adams HP, Bendixen BH, Kappelle LJ, Biller J, Love BB, Gordon DL, Marsh EE (1993). Classification of subtype of acute ischemic stroke. Definitions for use in a multicenter clinical trial. Stroke.

[17] Wessler BS, Thaler DE, Ruthazer R (2014). Transesophageal echocardiography in cryptogenic stroke and patent foramen ovale: analysis of putative high-risk features from the risk of paradoxical embolism database. Circ Cardiovasc Imaging.

[18] Tomasello F, Mariani F, Fieschi C (1982). Assessment of inter-observer differences in the Italian multicenter study on reversible cerebral ischemia. Stroke.

[19] Dawson J, Lamb KE, Quinn TJ, Lees KR, Horvers M, Verrijth MJ, Walters MR (2009). A recognition tool for transient ischaemic attack. QJM.

[20] Sganzerla P, Rondi M, Pavone A, Aiolfi E, Facchinetti A, Funaro A, Negrini P (2015). Clinical performance of the new gore septal occluder in patent foramen ovale closure: a single-center experience. J Invasive Cardiol.

[21] Knerr M, Bertog S, Vaskelyte L, Hofmann I, Sievert H (2014). Results of percutaneous closure of patent foramen ovale with the GORE septal occluder. Catheter Cardiovasc Interv.

[22] Freixa X, Ibrahim R, Chan J (2013). Initial clinical experience with the GORE septal occluder for the treatment of atrial septal defects and patent foramen ovale. EuroIntervention.

[23] Messe SR, Gronseth G, Kent DM, Kizer JR, Homma S, Rosterman L, Kasner SE (2016). Practice advisory: Recurrent stroke with patent foramen ovale (update of practice parameter). Neurology.

